# Research trends on the relationship between Microbiota and Gastric Cancer: A Bibliometric Analysis from 2000 to 2019

**DOI:** 10.7150/jca.44126

**Published:** 2020-06-07

**Authors:** Tongchao Zhang, Xiaolin Yin, Xiaorong Yang, Jinyu Man, Qiufeng He, Qiyun Wu, Ming Lu

**Affiliations:** 1 Department of Epidemiology and Health Statistics, School of Public Health, Cheeloo College of Medicine, Shandong University, Jinan, Shandong, 250012, China.; 2 Clinical Epidemiology Unit, Qilu Hospital, Cheeloo College of Medicine, Shandong University, Jinan, Shandong, 250012, China.; 3 Clinical Research Center of Shandong University, Jinan, Shandong, 250012, China.; 4 Department of Nutrition and Food Hygiene, School of Public Health, Nantong University, Nantong, Jiangsu, 226001, China.

**Keywords:** Gastric Cancer, Microbiota, Bibliometric Analysis, Research trend

## Abstract

**Background:** Hundreds of studies have found that the microbiota contributes to the development of gastric cancer in the past two decades. This study aimed to access the research trends of microbiota and gastric cancer.

**Materials and Methods:** Publications from January 1, 2000 to December 31, 2019 were retrieved from the Web of Science Core Collection database and screened according to inclusion criteria. Different kinds of software, SPSS21.0, HistCite, VOSviewer, CiteSpace, and the online bibliometric analysis platform were used to evaluate and visualize the results.

**Results:** A total of 196 publications were finally identified, and the annual number of publications showed an increasing trend. These publications were from 44 countries and the USA showed its dominant position in publication outputs, H-index, total citations, and international collaborations. The journal of *Helicobacter* was the most productive journal. Correa P and Peek RM published the most papers, and the most productive institution was Vanderbilt University. The keyword of “*Helicobacter pylori*” ranked first in research frontiers and appeared earlier, and the keyword of “microbiota” began to appear in the past 3 to 5 years.

**Conclusion:** The annual number of publications would continue to grow. Besides the traditional *Helicobacter pylori* related researches, future research hotspots will focus on microbiota and its mechanism of action.

## Introduction

Worldwide, Gastric cancer (GC) is the fifth most common malignant tumor and the third leading cause of cancer death, with about 1,000,000 new cases and an estimated 783,000 deaths of GC in 2018 1. The high-incidence areas are concentrated in East Asia, East Europe, and South America 1, 2. Although the incidence of GC has declined in most parts of the world, the burden is still high as a result of population growth 3. The prevention and treatment of GC remains important. Recently, more and more studies find that the development of many diseases may be related to the dysbiosis of microbiome. Microbiota refers to collective microbial community that live in specific environments, including bacteria, fungi, viruses, and protozoa 4. Studies have revealed the association between microbiota and some chronic diseases such as asthma, allergic diseases, chronic immune-mediated inflammatory diseases, type 1 diabetes and obesity 5. Now, growing evidence suggests that the microbiota also contributes to the development of cancers 4.

*Helicobacter pylori* (*H. pylori*), a common part of the human stomach microbiota, is firstly observed to be associated with the development of GC 6-8. Thereafter, a growing amount of evidence has found that the virus infection, such as *Epstein-Barr virus* (*EBV*) 9 and* human papillomaviruses* (*HPVs*) 10, results in GC. With the development of sequencing and experimental technology, recent evidence suggests that the dysbiosis of the microbiome in oral 11, gastric 12, and gut 13 is associated with GC. Hundreds of studies on microbiota and GC have been published in the past two decades, however; few researches have summarized this topic from the perspective of bibliometric analysis and provided the developing trends of this research domain.

Bibliometric analysis is a statistics method based on the public literature database (such as Web of Science) to analyse and visualize the research trend 14. Bibliometric analysis not only provides quantitative and qualitative evaluation of the publication, but also provides the developing trends of a research domain. It can also present the most influential research quickly and accurately, which provides theoretical basis for further research 15. In addition, this information can also provide policy guidance to decision makers 16. Therefore, in this study, we performed a bibliometric analysis of the publications on microbiota and GC published from 2000 to 2019. According our results, we will provide an overview of the achievements and future research trends and hotpots in this research domain.

## Materials and methods

### Data sources and search strategy

In this study, we used the Web of Science Core Collection (WoSCC), the most famous and influential scientific literature database, to search relevant literature on 10 March 2020. The WoSCC database is often used in bibliometric analysis as it has a strict assessment of publications, which guarantees the high quality of the literature 17-19. Meanwhile, the WoSCC database is updated continuously and dynamically, and can also provide the most influential, relevant and reliable information 17-19. The search strategy was TS= ((((((stomach) OR (gastric))))) AND (((((cancer*) OR (tumour*) OR (tumor*) OR (carcinoma*) OR (neoplasm*))))) AND (((((Flora*) OR (Microbiota*) OR (Microbiome*) OR (Microflora*) OR (Bacteria*)))))).

### Screening criteria

**Figure 1:**
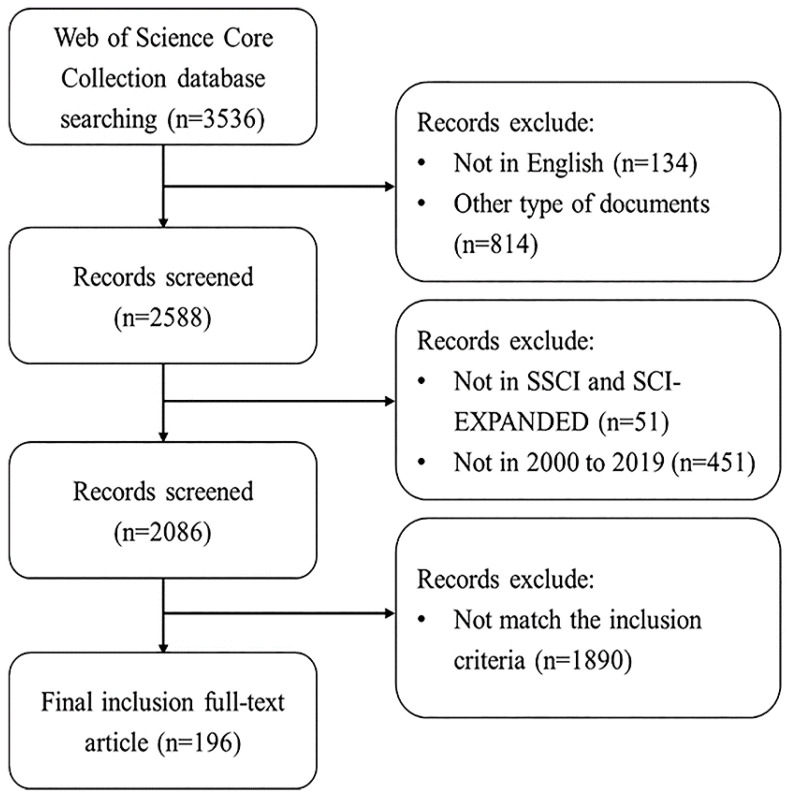
** F**lowchart of including and excluding publications.

The screening criteria are shown in Figure 1. After the initial search, we selected two authors to reviewed and screened the initially searched publications based on the following inclusion criteria: (1) The language of publication was “English”, (2) the publication type was “article”, (3) The publication came from Citation Index Expanded (SCI-E) and Social Sciences Citation Index (SSCI) database of WoSCC, (4) The timespan was selected between 2000 and 2019, (5) For the research content of the selected publication, the subjects of the publication were GC patients (including GC patients, preoperative and postoperative GC patients), animal model of GC, and cell model of GC, and the research content must simultaneously evaluate the correlation between the subjects and microbiota (including *H. pylori* or viruses). In order to avoid the bias caused by the daily updates of database, all searches and data collection were completed within the same day. According to the inclusion criteria, 196 publications were finally included in our study.

### Data preparation and information

The inclusive publications were downloaded and exported into different file formats for analysis. Analysis indicators included publication number, average citation of per publication, countries, institutions, journals, keywords, authors, Hirsch index (H-index, defined as the number of papers with citation number ≥h) 20, and the 2018 impact factor (IF). We also introduced the Bradford's law to describe and discover the “core journals”. The Bradford's law is defined as that if journals are arranged in order of decreasing productivity of publications and divided into three groups with the same number of publications, the number of journals in each group will be as 1:n:n².

### Statistical analysis

We used SPSS 21.0 software to analyze the trend of the publications according to the year. The logistic regression model, 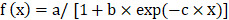, was used to curve fitting the cumulative number of articles 21. The growth inflection point of the curve (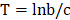) was the point when the article growth rate was the biggest, that was, from positive growth to negative growth 21. In this regression formula, x represented the particular year, and the f(x) represented the cumulative number of article by that year.

The different formats of the download file were imported into the HistCite, VOSviewer, CiteSpace, and Bibliometric analysis platform (https://bibliometric.com) for analysis. HistCite software (version 2.0, HistCite Software LLC, New York, USA) was used to count the indicators of country, journals, authors and institutions 22. VOSviewer (version 1.6.6; Leiden University Center for Science and Technology Studies, Leiden, Netherlands) was used to visualize bibliometric networks such as co-authorship and keywords analysis 23. We also used the software of CiteSpace (invented by Professor Chaomei Chen 24) to visualize the research trends of keywords. Furthermore, the online bibliometric analysis software (https://bibliometric.com/) was used to analyze the international collaboration between countries.

## Results

### 1. Global publications and citations

**Figure 2:**
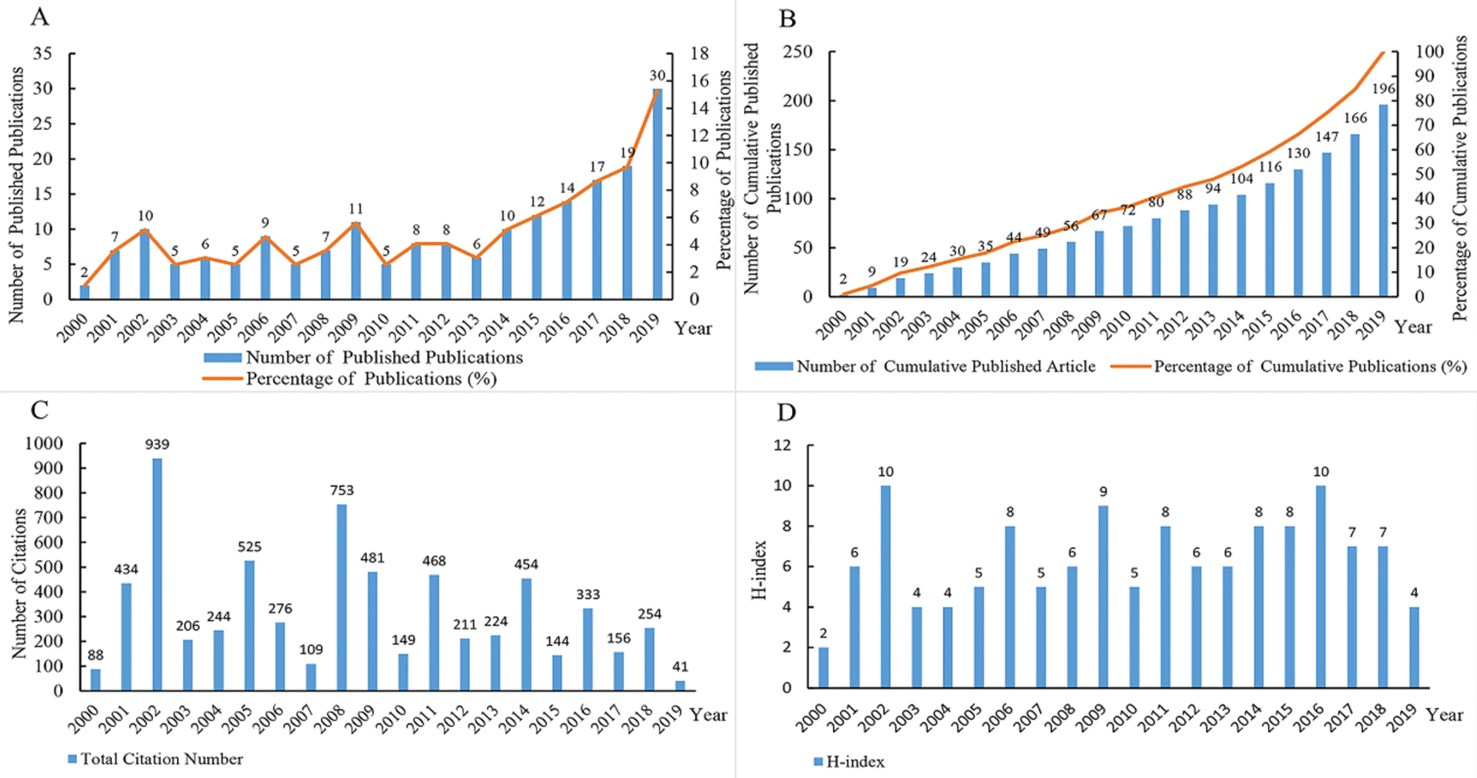
Global number of publications, number of citations, and H-index of publications in the field of microbiota and gastric cancer from 2000 to 2019. (A) Annual number of the published publications and its percentage; (B) Number and percentage of the annual cumulative published publications; (C) Annual citation number of the publications; (D) Annual H-index of the publications.

A total of 196 publications were published within the survey period, their attributes are presented in Figure 2. A growth trend in publication number was observed (form 2 in 2000 to 30 in 2019), and with 129 articles published in recent 10 years (account for 65.82% of the total publications, Figure 2A). The growth trend of the annual cumulative number of publication was consistent with the *S*-type growth curve model, and the formula was f(x) = 641.395/ [1+41.365×exp (-0.142×x)]. From the growth curve of the formula, it could be predicted that around 2026 might be the year with the highest publication growth rate (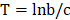). After 2026, the growth rate would decrease, however, the cumulative number of publication would continue to grow (Figure 2B). To the search date, these publications have been mainly cited for 6,022 times, and the average number of citations for these publications was 30.72. The relative high cited year (>300 times) was 2002 (939 times), 2008 (753 times), 2005 (525 times), 2009 (481 times), 2011 (468 times), 2014 (454 times), 2001 (434 times), and 2016 (333 times). Since the year 2017, 2018, and 2019 were close to the data collection time (10 March 2020), the citation number in the past three years were a little lower (Figure 2C). The H-index of these publications was 43, and the highest H-index year were 2002 and 2016 (Figure 2D).

### 2. Contributions of top 10 productive countries

**Figure 3:**
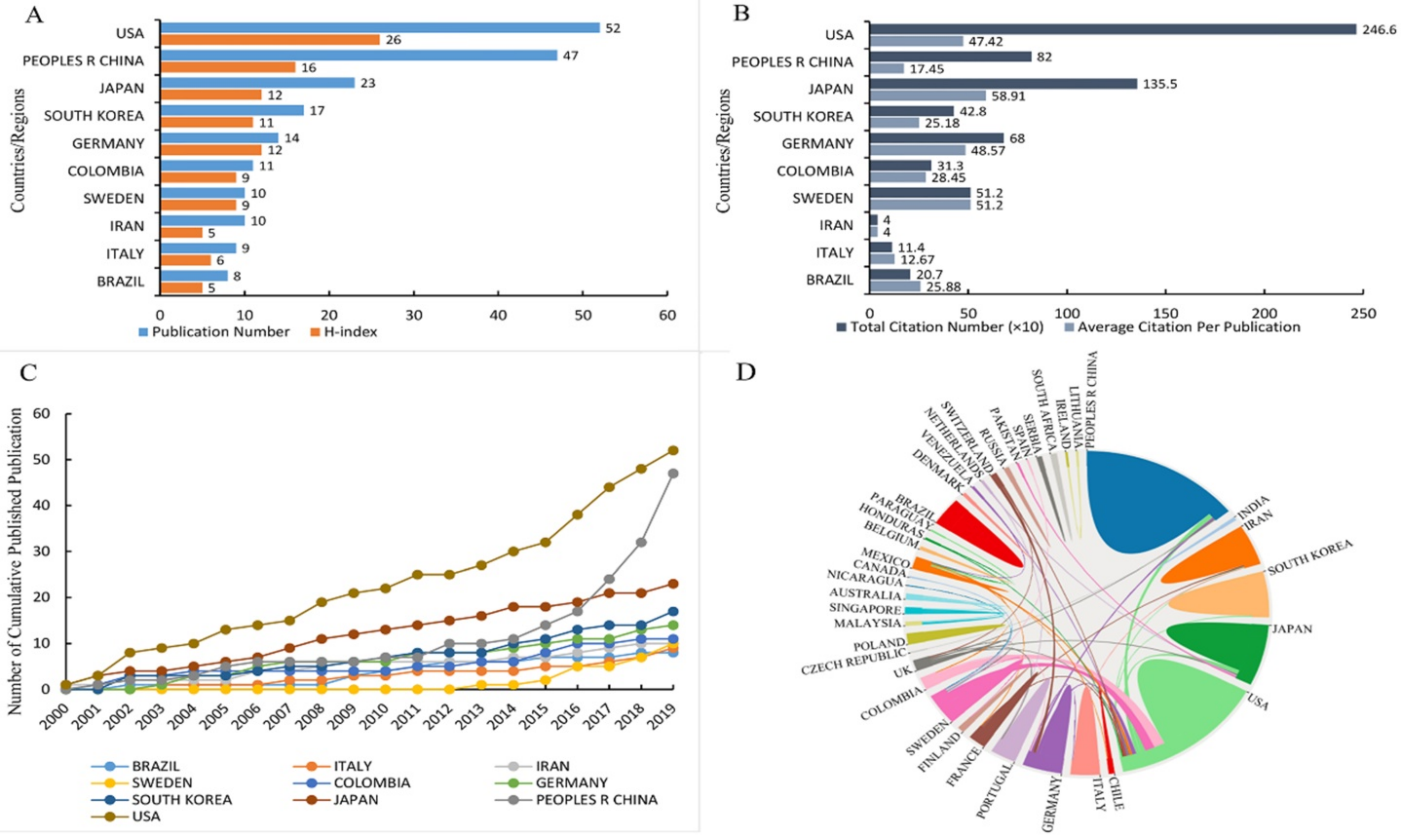
Top 10 productive countries related to microbiota and gastric cancer research from 2000 to 2019. (A) Number of publications and H-index; (B) Total number of citations and average citations of per publication; (C) Number of the cumulative publications in various countries; (D) International collaboration between countries. The countries were labeled using different colors and the links represented international collaborations.

The number of countries participating in the publication of these publications was forty-four. The USA, published 52 articles, was the most productive country, followed by China (47), Japan (23), South Korea (17), Germany (14), Colombia (11), Sweden (10), Iran (10), Italy (9), and Brazil (8) (Figure 3A). In the aspect of H-index, the USA (26), China (16), Japan (12), and Germany (12) ranked the top 3 highest H-index countries. Meanwhile, the H-index of the USA was higher than other countries (Figure 3A). The first 3 countries of total number of citation were the USA (2,466 times), Japan (1,355 times), and China (820 times) (Figure 3B), while the Japan (58.91 times), Sweden (51.20 times), and Germany (48.57 times) were the top 3 countries with the highest average number of citation (Figure 3B). The analysis of the cumulative number of publication in different countries within the survey period showed that the USA was a country with the most publications, followed by China and Japan. Moreover, the cumulative number of publication in China increased sharply since 2014, from 11 in 2014 to 47 in 2019 (Figure 3C). The USA participated in international cooperation most frequently, followed by UK, Sweden, and Mexico. China cooperated more closely with the USA, UK, and Germany, while the Brazil hardly cooperated with other countries (Figure 3D).

### 3. Article distribution among leading journals, institutions, and authors

The 196 inclusive publications were totally published in 105 journals. The top 10 productive journals have totally published 70 publications, accounting for 35.71% of the total articles. Based on Bradford's law, we discovered 12 journals and defined them as “core journals” in this study field (Table 1). The most productive journal was *Helicobacter* (11), followed by *Sci Rep* (8), *Gastroenterology* (7), *Int J Cancer* (7), *Plos One* (6), *Cancer Res* (5),* Gut* (5), *Digest Dis Sci* (5), *P Natl Acad Sci USA* (4), *J Immunol* (4), *Infect Immun* (4), and *Am J Gastroenterology* (4). The *P Natl Acad Sci USA* (784 times), *Gastroenterology* (591 times), and *Cancer Res* (431 times) were the first 3 journals with the highest total number of citation; while the first 3 journals of average citation per paper were* P Natl Acad Sci USA* (196.00 times), *Cancer Res* (86.20 times), and *Gastroenterology* (84.43 times). *Helicobacter* (8), *Gastroenterology* (7), *Int J Cancer r* (6), and *Sci Rep* (6) ranked the first 3 highest H-index journals; *Gastroenterology* (19.233),* Gut* (17.943), and *Am J Gastroenterology* (10.241) occupied the first 3 journals with the highest IF (2018) (Table 1).

**Figure 4:**
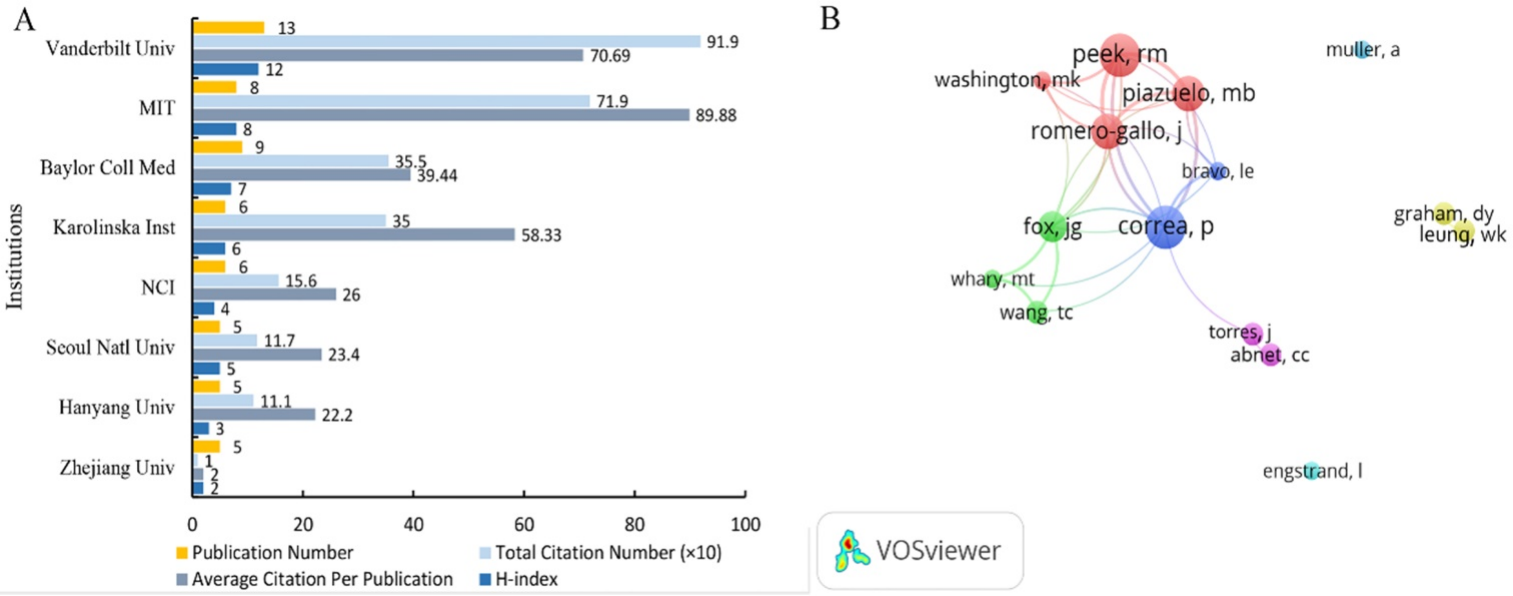
Article distribution among institutions and authors in the field of microbiota and gastric cancer from 2000 to 2019. (A) Publication number, total citation number, average citations of per publication, and H-index of the top 8 reproductive institutions; (B) Co-authorship among 15 reproductive authors. Dots represented authors and larger dot indicated higher number of publications, the clusters were labeled using different colors and the links represented author collaborations.

Furthermore, we evaluated the most productive institutions and authors in our study. Vanderbilt University, published 13 publications, was the most reproductive institution, followed by Baylor College of Medicine (9), Massachusetts Institute of Technology (8), and other institutions (Figure 4A). The Vanderbilt University was the institution with the highest total number of citation (919 times), while the Massachusetts Institute of Technology was the institution with the highest average citation (89.88 times) (Figure 4A). The first 3 highest H-index institutes were the Vanderbilt University, Massachusetts Institute of Technology, and Baylor College of Medicine (Figure 4A). In total, 1262 authors participated in these publications, with an average of 6.44 numbers of authors per publication. Based on the co-authorship analysis performed with VOS Viewer, we further defined “core author” as one who had published at least 4 papers, with the paper cited at least 100 times. Totally, there were 15 authors included in the final analysis, and some dominant research teams were found. Correa P and Peek RM was the most productive researcher with 10 publications, followed by Piazuelo MB (8), Romero-Gallo J (8), and other researchers (Figure 4B). Moreover, Correa P, Torres J, Abnet CC, Bravo LE, Peek RM, Washington MK, Romero-Gallo J, Piazuelo MB, Fox JG, Wang TC, and Whary MT cooperated closely, while they did not cooperated frequently with Muller A, Engstrand L, Graham DY, and Leung WK (Figure 4B).

### 4. Keywords visualization

**Figure 5:**
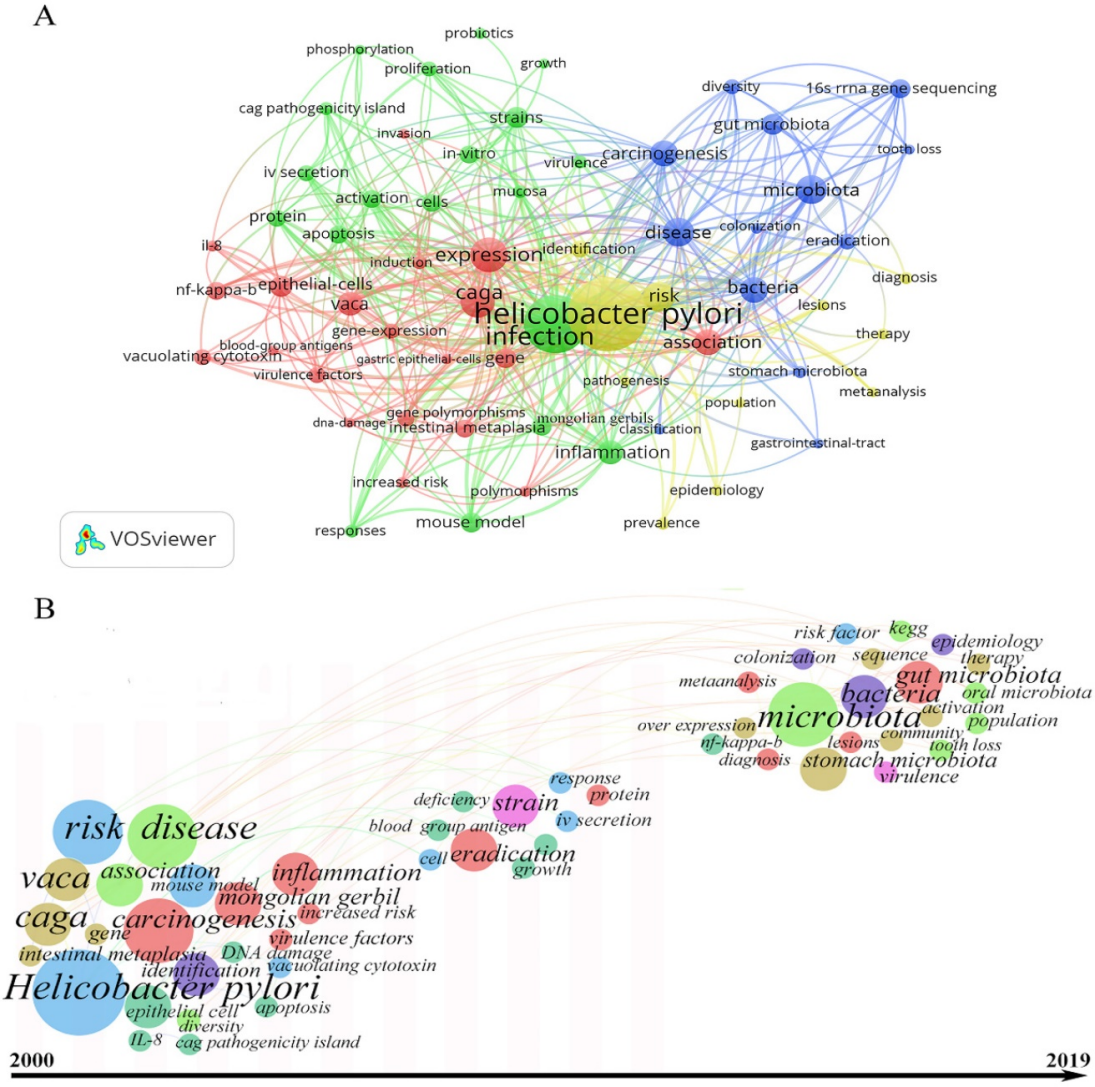
Keywords visualization related to microbiota and gastric cancer research from 2000 to 2019. (A) Network visualization of the keywords; (B) Time-based visualization of keywords variation. Dots represented keywords and larger dot indicated higher occurrence frequency of keywords, the clusters were labeled using different colors and the links represented the co-occurrence of keywords.

The software of VOSviewer and CiteSpace was used to visualize the occurrence frequency and time trend of keywords. The keywords such as “gastric cancer”, “stomach cancer”, “cancer”, and “carcinoma” were excluded; meanwhile, we unified the keywords such as “*Helicobacter pylori*” to get better perspective. According to the principle that the frequency of the keywords was at least 5 times, we totally introduced 63 keywords into analysis. We found that keywords of “*Helicobacter pylori*” and “infection” were the most prominent keywords, and most co-existed with “caga”, “vaca”, “expression”, “association” and “risk”. The inclusion keywords could be divided into four clusters: (1) epidemiology, diagnosis and therapy, (2) animal and in vitro cell models, (3) *H. pylori* and microbiota, and (4) virulence factors, immunological mechanism and gene expression/ploymorphisms (Figure 5A). Meanwhile, the analysis results of development of keywords over time showed that “*H. pylori*”, “carcinogenesis”, “caga”, “vaca”, and “gene” first appeared in this research field, followed by “inflammation”, “strain”, and “eradication”. Finally, “microbiota”, “stomach microbiota”, “gur microbiota”, “oral microbiota”, and “sequencing” began to appear in the past 3 to 5 years (Figure 5B).

## Discussion

In this study, by the use of visualization software, we aimed to explore the research trends and hotspots in the research field of microbiota and GC from 2000 to 2019. Our results suggested that the number of publication showed a growth trend and 2026 might be the year with the highest publication growth rate. This research field may remain a hotpot in the next few years. Based on the analysis of citation number of the publication and H-index, we found that publications with the highest citation and H-index were located in 2002. Therefore, the publications from 2002 had an important role in the research field of microbiota and GC, and should be carefully studied. Since the year 2017, 2018 and 2019 were close to the data collection time (10 March 2020), the citation number and H-index in the past three years were a little lower. However, the publications in recent years will be more cited.

The publications not only presents a dynamic time trend varying with the years, but also shows differences among different countries. The USA, China, and Japan ranked the top 3 productive countries, accounting for 62.24% of the total publications. The USA shows its dominant position in this research field, reflecting in publication outputs, H-index, total citations, and international collaborations. Notably, compared with the gradual increase of the cumulative number of publication in the USA and Japan, publications of China have increased dramatically after 2014. This increased phenomenon suggests that China is developing rapidly in this research field and is gradually developing towards high-quality research. However, at this stage, the number of citations in the Chinese publications is relatively lower, which may be due to the high proportion of the publications in the past five years (68.37%). We can also conclude that the Japan is one of the countries with the most citation of publication, suggesting its domain impact in this research field.

Among the top 8 productive institutions, 50.00% are located in the USA, indicating the higher quality of articles published by the USA institutions. Research institutions, such as Vanderbilt University, are relatively mature in this research field and can be considered as an important institution for collaboration and further learning. Correa P, Peek RM, Piazuelo MB, Romero-Gallo J, and Fox JG have published more publications and collaborated closer, and can be regarded as the leaders in this research field. Meanwhile, the journal distribution of the publications was concentrated in the first 12 journals. These journals can be divided into four categories: gastrointestinal cancer related journals, helicobacter professional journals, immunology mechanism journals, and comprehensive journals. The average citation of the journal publication was higher than the IF of the journal. This demonstrates that microbiota and GC is a new important research area, and the publications will be cited more frequently.

Results of keywords co-occurrence analysis first revealed that the inclusion keywords were divided into four clusters, representing (1) epidemiology, diagnosis and therapy, (2) animal and in vitro cell models, (3) *H. pylori* and microbiota, and (4) virulence factors, immunological mechanism and gene expression/ploymorphisms. These four clusters fully demonstrate the main concentrated aspects of the research field. We can recognise that this research field is still in the developing stage, admittedly, it will develop rapidly in the future. Then, from the frequency of occurrence of keywords, we found that “*Helicobacter pylori*” and “infection” were the most prominent keywords, and most co-existed with “caga”, “vaca”, “expression”, “association” and “risk”. These keywords appeared in earlier stages of the research field (mainly risk factors researches), and gradually extended to the mechanism research, such as immunological mechanism (the keywords such as “inflammation”, “strain”, and “eradication” appeared). This suggests that *H. pylori* is the most widely researched bacteria in this research field.

*H. pylori* is identified as a major pathogenic factor for chronic gastritis, dyspepsia, peptic ulcer, and GC 25, 26. From our results and previous researches, we can conclude the biology of *H. pylori* inducing GC.* H. pylori* secretes a variety of proteins (such as urease, carbonic anhydrase, Lewis antigen, VacA, CagA) to establish a suitable microenvironment for colonization and to induce pathogenic effects of inflammation 26. The mechanisms of GC induced by *H. pylori* are complicated. *H. pylori* can cause GC by inducing inflammation, DNA damage and gene mutation, epigenetic modification, cell apoptosis and autophagy, and other unknown mechanisms 25. Therefore, it can be predicted that the related researches of *H. pylori* and GC will continue to be a research hotspot.

Meanwhile, we analysed the development of keywords over time to reveal the frontiers and hotspots in microbiota and GC research. The results showed that the keywords such as “microbiota”, “stomach microbiota” “gur microbiota”, “oral microbiota”, and “sequencing” began to appear in the past 3 to 5 years. This supports our view that the development trend of this research field is from previous single bacteria research (such as *H. pylori*) to the microbiota research. This research trend is closely related to the development of sequencing and experimental technology in recent years. By the wide use of 16S rRNA sequencing technology, other members of microbiota of the human stomach, such as *Proteobacteria*, *Firmicutes*, *Actinobacteria*, *Bacteroidetes*, and *Fusobacteria* phyla, were identified 27-29. The mucosal microbial compositional changes and the special bacterial taxas (*Peptostreptococcus stomatis*, *Streptococcus anginosus*, *Parvimonas micra*, *Slackia exigua* and *Dialister pneumosintes*) play a potentially important role in GC progression, and these bacterial taxas are members of the oral microbiota 30. Ferreira RM's study revealed that GC microbiota was characterized by decreased abundance of Helicobacter, while other members of bacterial taxas such as *Citrobacte*r, *Clostridium*, *Lactobacillus*, *Achromobacter*, *Rhodococcus*, and *Phyllobacterium* were significantly enriched 28. The composition of gastric microbiota is similar to that of oral microbiota in phylum-level taxonomical profiles when *H. pylori* is removed 31. Therefore, some researches have begun to explore the association between oral microbiota and GC, and to apply the oral saliva microbiota for screening suspected GC patients 32, 33.

The microbiota may induce carcinogenesis in the following potential ways. First, changes in diversity and abundance of microbiota (which is termed dysbiosis) may be associated with the occurrence of GC 34. Gunathilake M's case-control study conducted in Koreans found that the Shannon index in the controls was significantly higher than in the GC patients 35, Ferreira RM's study also found that the microbial diversity of GC patients had significantly decreased 28. Second, virulence factors of bacteria may contribute to the development of GC. *H. pylori* exerts its carcinogenic effect mainly through two virulence factors: VacA and CagA 36. The virulence factors of Fusobacterium nucleatum (such as FadA and Fap2) play important roles in biofilm formation, tumor cell attachment, and invasion 37, 38. The bacteria and its virulence factors may cause carcinogenesis by inducing chronic inflammation and altering the microenvironment, immune response and regulation, and interfering with signal transduction pathway 39. Further studies are still needed to validate and explore the mechanisms. Moreover, the products of microbial metabolism also become an important factor in carcinogenesis. Some nitrosating bacteria, such as *Escherichia coli*, *Lactobacillus*,* Nitrospirae*, *Clostridium*,* Veillonella*, *Haemophilus*, and *Staphylococcus* 36, could convert dietary amines/nitrogen compounds in stomach to carcinogenic N-nitroso compounds (NOCs) 36, 40. Oral microbiota may increase the gastrointestinal cancer risk by increasing alcohol and smoking-related carcinogenic metabolites 39.Therefore, the detection of microbial metabolism products will be a new research hotpot in revealing the mechanism of microbiota and GC.

Our study also had some limitations, which should be taken into when interpreting the results of our study. First, the publications are only derived from SCI-E and SSCI of WoSCC database, which might lead to incomplete literature searches. The WoSCC database has a strict assessment of publications, which guarantees the high quality of the literature 17-19. Meanwhile, the WoSCC database is updated continuously and dynamically, and can also provide the most influential, relevant and reliable information 17-19. Therefore, the WoSCC database is often used in bibliometric analysis and is built for this type of analysis 17-19, 41-43. Second, we only introduced the English publications into our analysis, however, we have included all the important and classic publications into our analysis. Last, a certain bias in the selection of publications should not be excluded, although we selected two people to review and screen the initially searched publications. Meanwhile, we developed a series of strict screening principles, therefore; a lot of non-compliant documents were filtered out. Although we have some limitations, we still reveal the future research trends and hotspots in this research filed to some extent.

In conclusion, the annual number of publications on microbiota and GC have grown rapidly in the past two decades and will continue to grow. The USA is the leading country in this research field. China also achieves some important research results and plays a certain role in promoting the development of this research filed. Besides the traditional *H. pylori* related researchers, future research hot spots will domain in microbiota and its mechanism of action.These results provide a new perspective for the study of microbiota and GC, which may have a beneficial effect on the further etiological study, diagnosis, and treatment of GC.

## Figures and Tables

**Table 1:** Top 12 leading journals related to microbiota and gastric cancer research from 2000 to 2019

Journal title	Records	Total citations	Average citation per paper	H-index	IF (2018)
*Helicobacter*	11	252	22.91	8	3.352
*Sci Rep*	8	236	29.50	6	4.011
*Gastroenterology*	7	591	84.43	7	19.233
*Int J Cancer*	7	223	31.86	6	4.982
*Plos One*	6	99	16.50	5	2.776
*Cancer Res*	5	431	86.20	5	8.378
*Gut*	5	357	71.40	5	17.943
*Digest Dis Sci*	5	59	11.80	4	2.937
*P Natl Acad Sci USA*	4	784	196.00	4	9.580
*J Immunol*	4	217	54.25	4	3.160
*Infect Immun*	4	209	52.25	4	4.718
*Am J Gastroenterology*	4	121	30.25	4	10.241
